# Information Disorder Syndrome and its Management

**DOI:** 10.31729/jnma.4968

**Published:** 2020-04-30

**Authors:** Nirmal Kandel

**Affiliations:** 1 Member, Nepal Medical Association, Kathmandu, Nepal

**Keywords:** *disinformation*, *information disorder*, *misinformation*

## Abstract

Many of us may be unknowingly suffering from information disorder syndrome. It is more prevalent due to the digitized world where the information flows to every individual's phone, tablet and computer in no time. Information disorder syndrome is the sharing or developing of false information with or without the intent of harming and they are categorized as misinformation, disinformation and malinformation. The severity of the syndrome is categorized into three grades. Grade 1 is a milder form in which the individual shares false information without the intent of harming others. Grade 2 is a moderate form in which the individual develops and shares false information with the intent of making money and political gain, but not with the intent of harming people. Grade 3 is a severe form in which the individual develops and shares false information with the intent of harming others. The management of this disorder requires the management of false information, which is rumor surveillance, targeted messaging and community engagement. Repeated sufferers at the Grade 1 level, all sufferers from grade 2 and 3 levels need psycho-social counseling and sometimes require strong regulations and enforcement to control such information disorder. The most critical intervention is to be mindful of the fact that not all posts in social media and news are real, and need to be interpreted carefully.

## INTRODUCTION

Britannica defines propaganda as “dissemination of information—facts, arguments, rumors, half-truths, or lies—to influence public opinion”.^1^ There is a long history of the use of propaganda starting from the 16th century, some believe it to be as early as 500 BCE.^2^ The use of propaganda was mostly for political purpose and faith-based institutions in the past, and it would take a lot of time and resources to establish and disseminate. With a digitized world, there are a lot of information disorders including propaganda, and it is not limited to politics. The world is super connected through the internet, social media and search engines. Ideally, access to real-time information with a click would have brought lots of positive changes. But this is not a case and what we are observing now is that the information ecosystem is dangerously polluted and dividing us rather than connecting us.^3^

We have sham websites, fake social media accounts, click farmhouses to manipulate the media platform with recommendations and trending of hashtags and communities creating and disseminating fake news, rumors, hoaxes, videos, memes and social feeds. To understand, who owns and how these are managed and manipulated is very difficult in this complex information ecosystem.^3^ Different groups have their vested interests and are using these tools for their gain, ignoring the impact in public. Still, many of us are part of it knowingly or unknowingly. We all are providing a fertile ground to these groups by sharing and re-sharing posts in social media without understanding the consequences, believing in fake news and sham websites and favoring one's prejudiced beliefs and faiths while slandering others.^4^

### INFORMATION DISORDER SYNDROME

Distorting facts, manipulating information, sharing information without understanding the consequences, vilifying others' beliefs and faiths, and running behind propaganda and fake news with or without vested interest is some of the kind of a disorder. Claire Wardle and Hossein Derakhshan first researched and published about the different types of information disorders,^4^ and categorized three kinds of information disorders: a) Disinformation, b) Misinformation and c) Malinformation. Humans are considered the wisest and most intelligent living beings, who have the capability of differentiating right and wrong. Despite these strengths, many are influenced by these information disorders either knowingly or unknowingly. And I am calling this phenomenon as an information disorder syndrome. Anyone who develops, shares or part or whole of the disinformation, misinformation or malinformation is suffering from the information disorder syndrome.

Information disorder syndrome applies to all cases of false information and not limited to a specific topic. The paper tries to cite examples from the ongoing outbreak of false information and others.

Disinformation is content that is intentionally false and designed to cause harm. It is motivated by three distinct factors: to make money (financial); to have political influence, either foreign or domestic (political); or to cause trouble for the sake of it (psychological or social). When disinformation is shared, it often turns into misinformation and keeps on circulating.

During any disease outbreak, the claim of a drug or vaccine being developed and advertised online could be false information to make money.^5^ Predatory conferences on the Zika Virus and Ebola pandemic are other examples of wrongly capitalizing on emergencies. An outbreak has economic and political consequences and manipulating information like trolling against a country, organization and individuals are a few examples of disinformation.^6^ Messages on the internet about false claims on the origin of a virus and causes of spread from a group of conspiracy theorists and blaming local scientists led a group of scientists to come together to manage this disinformation.^6^ “The rapid, open, and transparent sharing of data on the COVID-19 outbreak is now being threatened by rumors and misinformation around its origins,” warn authors in a recent article in The Lancet.^6^

Misinformation is an untruthful content but the person sharing does not realize that it is false or deceptive i.e. they don't have the intent of harming others. Often a piece of disinformation is picked up by someone who does not realize it is false, and shares it with their networks, believing that they are helping. Psycho-social factors contribute to the sharing of misinformation.

For instance, misinformation on COVID-19 includes, a list is long such as, “viruses are scared of acid, use a cotton bud with strong vinegar/sesame oil and stick it inside your nose”; “stop wearing woolen clothes”, and “use eight pods of garlic for prevention”.^7,8^ There are also socio-psychological factors that create pressers to increase their followers or networks. Such people share disinformation or misinformation without realizing any consequences of their post or publication.^9^

An example of tampered or misinterpreted video or social media feed includes the spread of misinformation by a video of a Chinese woman biting into a bat, falsely suggesting it was shot in Wuhan and caused the outbreak. This video was actually from Palau in 2016 as part of a show on Palauan cuisine.^10^ Another video, which purported to show a collapsed building during the 2015 earthquake in Nepal, was actually from another conflict-affected country.

Malinformation is information that is shared with an intent to cause harm. Examples include the anti-vaxx-ermovement,^11^ and those who have made have false claims of sexual abuse and harassment. In some cases, such information maybe difficult to fact check or investigate. Malinformation often includes private information that is spread to harm a person or reputation.^12^ For example, people from certain regions demonize an individual country as spreaders of disease.^13,14^ Such falsehood only serves to stoke racism, racial discrimination, xenophobia, and related intolerance, needlessly straining our social fabric.^13^ On 24th January, a video went viral appearing to be of a nurse in Hubei province describing a far worse situation in Wuhan than purported by Chinese officials. The video claims that more than 90,000 people have been infected with the virus in China alone.^15^ The woman does not claim to be a nurse or a doctor in the video and that her protected suit and mask do not match the ones worn by medical staff in Hubei.^16^

### MANAGING INFORMATION DISORDER SYNDROME

A 2018 study from Massachusetts Institute of Technology found that “false news spreads more rapidly on the social network than real news does”.^17^ Newly available false news was more likely to be shared among people. Whereas false stories inspired fear, disgust, and surprise in replies, true stories inspired anticipation, sadness, joy, and trust. As such, it requires a systematic approach, as proposed below.

**Figure 1.**
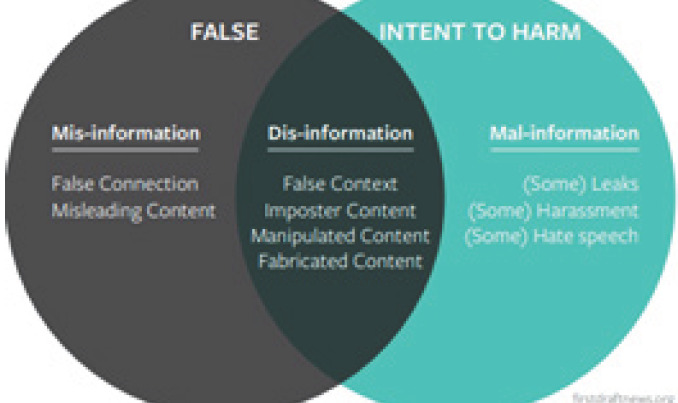
Types of information disorders.

Information disorder syndrome is categorized into three grades based on its level and use of false information with or without the intent of making money and causing harm. A few of the examples used above have been classified under the types of information disorders by Wardle C3 (Figure 1).

There are categories of all the different construction of misinformation (false connection and misleading content), disinformation (false context and imposter, manipulated and fabricated contents) and misinformation (some leaks, harassments, and hate speech) (Table 1).^18^

**Table 1:** Grading for information disorder syndrome.

Grading	Features (Definitions)	Types and examples	Management
		Misinformation	Key Strategies
Grade 1	Most people fall into this category and share information with or without understanding whether it is correct or not and the potential consequences.	1. Sharing fake news, social media feed, memes, etc.	1. Rumor surveillance
	Shares disinformation and malinformation but they are not the originators, nor they are benefiting from it.	2. Shares in virtual or physical groups	2. Targeted evidence-based messaging and community engagement
		3. Garlic cures COVID19,	3. Psycho-social counseling
		4. Facebook giving 10 million to 100 users, etc.	
**Misinformation and Disinformation**			
Grade 2	Shares or develops misinformation or disinformation with the intent of making money and political influence but not to harm people.	1. Predatory conferences on the emergencies,	1. Rumor surveillance
	They are either the originators of disinformation or have the capacity to capitalize benefits from existing misinformation/disinformation	2. Travel and hotel recommendation in search engines & social media with false information	2. Targeted evidence-based messaging and community engagement
			3. Psycho-social counseling
			4. Development or enforcement of regulatory mechanisms to control the disinformation
**Disinformation and Malinformation**			
Grade 3	Shares or develops misinformation or disinformation with the intent of harming people with or without the intent of making money.	Anti-vaxxer movement	1. Rumor surveillance
		False cases of abuse, rape or harassment, etc. (e.g. Depeche mode) (12)	2. Targeted evidence-based messaging and community engagement
			3. Psycho-social counseling
			4. Development or enforcement of regulatory mechanisms to control the disinformation and malinformation

### MANAGEMENT OF FALSE INFORMATION: RUMOUR SURVEILLANCE, TARGETED MESSAGING AND COMMUNITY ENGAGEMENT

Management of information disorder syndrome should start with the surveillance of the information itself. The best way to monitor this is to conduct rumor surveillance routinely with the support of social media, media platforms and tech companies. We can learn a lot about managing false during this emergency. International agencies are working closely with social media like Facebook, Pinterest, Twitter, Amazon, Google, YouTube, Weibo, Tiktok to tackle the spread of false information.^5,19^ For example, when users searched for the term coronavirus on Amazon, listings for face masks and vitamin C boosters showed up. Similarly, Vitamin C has also been listed as one of the fake cures for coronavirus.^5^

The next step, based on the findings of rumor surveillance and prioritization, is to target the sources of false information, with containment and mitigation measures like notification, removal of false information and raising awareness through active engagement. Taking an example of COVID-19, many governments, agencies and institutions are busting a lot of myths and rumors with evidence-based information which is also made available on social media channels (including Weibo, Twitter, Facebook, Instagram, LinkedIn, Pinterest) and website.^19^

Facebook is limiting the spread of false information about the coronavirus by removing “false claims or conspiracy theories”. It is using its existing fact-checkers to review and expose misinformation. Facebook has said that they would notify individuals who have shared or were trying to share information that had been flagged as false in relation to the current pandemic.^20^ They are focusing posts on “claims that are designed to discourage treatment” including social feeds about false cures such as the post advising Facebook users to “keep your throat moist” and avoid spicy food to prevent COVID-19. These feeds were shared over 16,000 times.^5^

Twitter mentioned that there were 15 billion tweets about coronavirus in the first four weeks of the year. It has launched a prompt that appears when users search for coronavirus or COVID-19, encouraging them to use official channels of WHO or Centres for Disease Control and Prevention - for information.^5^ When Twitter users search for coronavirus or COVID-19 a large headline with the title “Know the facts” appears. Monitoring and evaluating and analyzing twitter feeds' can provide a lot of information. Tweet analysis was also used in the past to monitor the Middle East Respiratory Syndrome.^21^

### PSYCHOSOCIAL COUNSELING

Psycho-social counseling may be necessary for every individual who is suffering from information disorder syndrome. Mostly for individuals at the Grade 1 level individual, targeted messaging and community engagement can help prevent and control information disorder. Those who are addicted to social media, and have excessive online activity, narcissism, and are at Grade 2 and 3, require psycho-social counseling. Cognitive-behavioral therapy (CBT) and motivational enhancement therapy are recommended, although there is no gold standard intervention.^22^ The most critical intervention is to be mindful of the fact that not all posts in social media and news are real. They need to be interpreted carefully. Managing the outbreak of information disorder syndrome at the individual level is challenging; this is where targeted evidence-based messaging is essential.

### REGULATION AND ITS ENFORCEMENT

Rumor surveillance, targeted messaging, and community engagement is only possible when it is limited to misinformation, and there is no intention of harm. When false information is spread to harm others, then there is a need for regulations and enforcement. A few such initiatives are necessary to tackle people and groups, especially those who are suffering from Grade 2 and Grade 3 levels of information disorder syndrome, who are harming others. The deadly measles outbreak in Samoa infected over 4800 people and killed over 70, mostly young children.^23^ Subsequently, the Samoan Government arrested an anti-vaxxer for spreading fake news during the outbreak in Samoa.^23^ Recently, the government of Nepal arrested a man who spread a rumor of deaths due to COVID-19 in one of the private hospitals and punished him under the law of cybercrime.^24^

Earlier this month, a Malaysian journalist was charged for making statements that could cause public alarm over social media posts related to the virus, including one in which she raised concerns about the arrival of Chinese tourists on a cruise ship. Officials have warned that false information about the virus could be “inflammatory” in the Muslim-majority, multi-ethnic Malaysia, where race and religion are considered sensitive topics.^25^
